# ‘She’s surrounded by everybody, but yet seen by nobody.’ Parents’ experiences and priorities for neonatal developmental follow-up care

**DOI:** 10.1136/bmjpo-2026-004772

**Published:** 2026-08-07

**Authors:** Claire Marcroft, Anna Purna Basu, Niina Kolehmainen, Richard Thomson, Catherine Exley, Parent Advisory Group, Megan Tinning

**Affiliations:** 1 Newcastle Neonatal Service, Newcastle Upon Tyne Hospitals NHS Foundation Trust, Newcastle upon Tyne, England, UK; 2 Population Health Sciences Institute, Newcastle University, Newcastle upon Tyne, UK

**Keywords:** Qualitative research, Neonatology, Infant, Health services research, Child Health

## Abstract

**Background:**

Infants born very preterm (<30 weeks’ gestational age) are at increased risk of long-term developmental challenges. Monitoring health and development after discharge home is an essential aspect of neonatal follow-up care; however, there is limited understanding of how parents and carers experience this care with their child or how their own needs are addressed.

**Methods:**

This study aimed to understand and evaluate parents’ experiences of neonatal developmental follow-up. Participants were recruited purposively from three diverse National Health Service providers in England. Semi-structured qualitative interviews were conducted, and data were generated and analysed using a Constructivist Grounded Theory approach. Data collection and analysis occurred concurrently, supported by memoing, reflexivity and peer debriefing. Initial and focused coding led to the iterative development of categories.

**Results:**

Between January 2023 and October 2024, 24 interviews were conducted with parents of infants born before 30 weeks of gestation. Three categories were generated from the data analysis: parent support needs, disintegrated care and healthcare professional (HCP) experience and behaviours.

**Conclusion:**

Parents highlighted that their psychological support needs were unmet during their child’s neonatal developmental follow-up, and they found navigating healthcare services after discharge home challenging. Changes to care priorities are required, emphasising the development of integrated systems across service providers and geographical areas, and training for universal healthcare providers.

**WHAT IS ALREADY KNOWN ON THIS TOPIC**

- Children born extremely prematurely are at risk of lifelong health and developmental challenges. Limited attention has been given to post-discharge pathways, and parents’ perspectives on neonatal developmental follow-up have not been well explored.

**WHAT THIS STUDY ADDS**

- Parents understand developmental follow-up as a process that focuses on their child’s health, growth and development and involves interactions with a diverse range of healthcare professionals from different service providers. Uncertainty about clinical service accountability for care contributes to disintegrated care.
- Parents caring for preterm-born infants prefer to have early diagnostic confirmation of neurodevelopmental conditions associated with prematurity and highlight that their psychological support needs are unmet.

**HOW THIS STUDY MIGHT AFFECT RESEARCH, PRACTICE OR POLICY**

- Clinical guidelines with a systems perspective need to be developed to optimise the use of the different services involved in growth, health and developmental follow-up for children born very preterm, including parental and family well-being.
- Increased training and expertise are needed within universal child health services to enable professionals to deliver effective care.

## Introduction

 Preterm birth and perinatal brain injury can have lifelong effects on infants, their families and society.^1 2^ Although survival rates for infants born at the earliest stages of gestation have increased, there has been no corresponding improvement in rates of neurodevelopmental impairment.^3^ Health and developmental difficulties may emerge at different points in a child’s development, making neonatal follow-up services crucial for comprehensive care.^4^

Recommendations for neonatal developmental follow-up have been published worldwide.^5–8^ In the UK, the need to improve neonatal follow-up services was highlighted in the House of Lords report ‘Preterm Birth: Reducing Risks and Improving Lives’.^9^ The UK guideline recommends that infants born before 30 weeks’ gestation and those with additional risk factors for developmental challenges should have their development monitored until (corrected) age 2, including a face-to-face developmental assessment at 24 months.^5^ For those born before 28 weeks, an additional assessment at age 4 is recommended to provide appropriate support and interventions in preparation for school. The guideline emphasises that enhanced developmental support and surveillance should be provided as an integral part of neonatal services, working in collaboration with local health services.

In the UK, neonatal care is organised through Operational Delivery Networks (ODNs), which are collaborative, multi-service networks that aim to ensure infants receive care in the most suitable locations for their medical needs.^10^ Access to these services is determined by several factors, such as the child’s medical needs and their home location. Despite this, there has been greater focus on developing policy on pathways of care preceding birth and during acute inpatient care, with limited attention to family priorities for care after discharge home. This ODN service structure offers a unique opportunity to establish coordinated post-discharge care pathways for infants and their families by facilitating collaboration across geographically diverse regions.

Previous studies have reported low parental self-confidence in managing their children’s ongoing medical needs at home, with many families feeling unprepared and unsupported.^11^ It is unclear ‘how’ parents experience neonatal follow-up care and whether current services offer the support they need or measure outcomes that matter to them.^12 13^

This research aims to understand parents’ experiences of neonatal developmental follow-up care and identify areas for improvement.

## Methods

### Study design

A qualitative study using Constructivist Grounded Theory (CGT)^14^ was used. Data were collected through semi-structured interviews, with concurrent analysis, supported by memo writing and meetings with research and parent advisory groups. According to the Enhancing the QUAlity and Transparency Of health Research (EQUATOR) guidelines, the study’s reporting follows the consolidated criteria for reporting qualitative research (COREQ) guidelines.^15^

### Setting

The research was conducted across three varied NHS neonatal service providers in Northern England, UK. These services were chosen to represent a range of service types, highlighting diversity in care levels, size, patient health needs, and organisational structure and policies covering both inpatient and follow-up care.

### Sampling and recruitment

Data from a UK survey of neonatal developmental follow-up clinical service provision^16^ guided purposive and theoretical sampling of study sites. Lead clinicians were contacted to assess their willingness to participate, and clinical gatekeepers identified eligible participants. Infant factors included variations in gestational age at birth, birth weight, age at interview (within the first 2 years), need for home oxygen and presence of comorbid conditions, eg, brain injury or chronic lung disease. Diversity also covered family factors, including civil and employment status, and home distance from the service provider.

### Inclusion criteria

Parents and family members over 16 years with a preterm infant(s) born before 30 weeks’ gestation.

### Exclusion criteria

Parents unable to provide written informed consent, those with infants diagnosed with or suspected to have genetic disorders, and those who could not speak English (due to lack of translation resources).

Participants were verbally informed about the study by a clinician during their hospital stay or neonatal follow-up appointment. Those who expressed interest received a study information sheet, and consent was obtained to share their contact details with the research team. The primary author (CM) contacted parents to discuss participation and obtain written consent, after which participants selected their preferred interview arrangement. Recruitment continued until theoretical sufficiency was reached,^17^ meaning that enough data had been gathered and analysed to develop conceptually coherent and well-supported categories reflecting a variety of infant and family circumstances.

### Ethical considerations

Ethical approval was obtained from the Health Research Authority (ref: 22/WA/0093) and from participating NHS services. Considerations focused on maintaining participant confidentiality through the use of pseudonyms. Participants received a £25 voucher as compensation for their time.

### Data collection

Participants joined a semi-structured interview conducted in person, via video (Microsoft Teams), or by telephone, which was conducted by CM and audio-recorded. A literature review and feedback from research and parent advisory groups informed the interview topic guide, which was piloted with two parent volunteers. The interview guide was used flexibly and regularly reviewed and updated during team meetings as the study progressed.

### Data analysis

**Figure 1:**
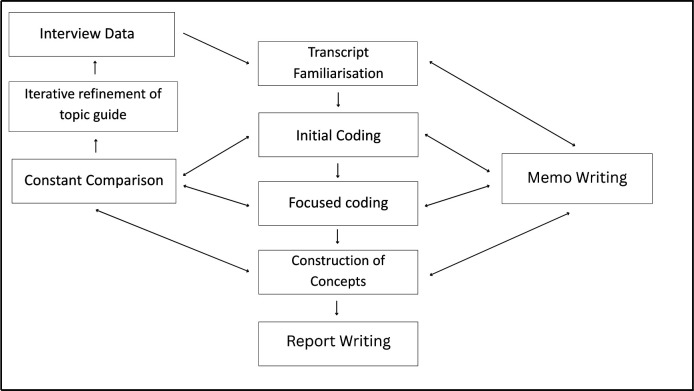
Constructivist Grounded Theory (CGT) process of data collection and analysis.^14^

Data were analysed iteratively using the constant comparison approach.^14^ Figure 1 illustrates the method’s stages, showing the researcher’s back-and-forth process as the data were simultaneously generated and analysed.

CM transcribed the initial two interviews to become familiar with the data. After this, the interview audio files were encrypted and transferred to an NHS-approved transcription service. CM anonymised the data, added handwritten notes, and uploaded them to NVivo 14 software (QSR International, 2023) along with written memos. CM conducted initial coding, applying labels to specific words and line segments and moving between data to link codes and processes, using memos to advance understanding.^14^ Discussions with the research team (CE, RT, AB and NK) and the parent advisory group (MT, RC and KS) occurred bimonthly and contributed to the analysis. Codes were discussed and iteratively adjusted to generate categories and confirm, extend or challenge emerging categories; data analysis also contributed to theoretical sampling. The analytic decisions supporting sample closure (sufficiency) were made when the defining characteristics of the categories were clearly expressed (had stabilised) and consistently appeared in parents’ narratives with confirmation from the parent advisory group supporting sample closure.

### Reflexivity

The primary researcher (CM), a female neonatal physiotherapist, works clinically in a neonatal intensive care unit with inpatient and developmental follow-up responsibilities. She conducted this research alongside her clinical role as part of an NIHR-funded doctoral fellowship. There was no pre-existing relationship between CM and participants. CM reflected on the potential impact of her experiences on the research¹⁴, with the research team and the parent advisory group, to enhance reflexivity.

### Patient and public involvement

Four parents of preterm infants contributed to the study’s design and development. This parent advisory group met throughout the study, advising on study design, participant information materials and data analysis.

## Results

### Participant characteristics

Between January 2023 and October 2024, twenty-four interviews were conducted with parents of babies born before 30 weeks’ gestation. Clinicians reported distributing expressions of interest and permission to share the contact information of 41 parents with the research team. Only one person declined participation. The sample was determined by theoretical sampling, considering factors related to infants and parents.

Table 1 provides general characteristics of these parents and their children and includes 19 singleton births and five sets of twins. Twenty interviews involved one parent (18 mothers and two fathers), while two interviews included both parents (four mothers and four fathers). Eighteen participants spoke only English; six spoke additional languages (including Polish, Filipino and Hindi), and four considered English a second language. Two parents had either a physical or a learning disability affecting their ability to care for their baby. The parent with a learning disability was supported by their partner during the interview (the partner was present, but not the focus of the interview). One participant was an adoptive parent, and all participants and their children were given pseudonyms. The mean interview duration was 45 min (range of 21–95 min).

**Table 1:** General characteristics of the parent participants interviewed

Participant	Parent gender	Child gender	Child GA at birth (weeks)	Child CA at interview (months)	Birth	Days of hospital care	Home oxygen	Site
01	F	M	28	27	Single	85	Y	1
02	F	F	27	18	Multiple	87	N	1
03	F	F+M	24	15	Multiple	142	Y	1
04	F	M	29	13	Single	43	N	1
05	F	M+M	25	29	Multiple	109	Y	1
06	F	F	26	20	Single	68	N	2
07	F+M	M	29	2	Single	57	Y	2
08	F+M	M+F	28	14	Multiple	78	N	2
09	F	M	29	15	Single	51	N	2
10	F	F	28	12	Single	52	N	2
11	M	M	29	17	Single	51	N	2
12	F+M	F+F	26	24	Multiple	102	N	1
13	M	M	28	15	Single	49	N	3
14	F	M	27	17	Single	99	Y	3
15	F	F	26	12	Single	56	N	2
16	F	M	27	32	Single	76	Y	3
17	F	M	28	25	Single	41	N	3
18	F	M+M	28	26	Multiple	86/108	Y	3
19	F	M	28	4	Single	73	Y	3
20	F	M	29	13	Single	56	N	1
21	F	M	24	3	Single	119	Y	1
22	F+M	F	27	12	Single	84	Y	2
23	F	F	24	5	Single	117	Y	3
24	F	F	29	8	Single	49	N	3

CA, corrected age; GA, gestational age.

Most infants and their families (71%) received medical care from two or more inpatient neonatal services before being discharged home (table 2).

**Table 2:** Number of inpatient services experienced before discharge home

Number of inpatient services	Number of infants and families
1	7
2	12
3	5

Interviews were conducted during the child’s first 2 years of life. Table 3 presents the distribution of the child’s corrected age at the time of the interview.

**Table 3:** Corrected age of child at time of interview with parents

Child’s corrected age at the time of the interview	Number of interviews with parents
0–6 months	4
7–12 months	5
13–23 months	9
24–32 months	6

Parental experiences focused on the infant’s health (including weight, weaning from oxygen and medications) and developmental progress. They included components beyond the scope of the NICE guidelines,^5^ such as interactions with healthcare professionals (HCPs) across multiple organisational boundaries. This included: universal health service providers, such as health visitors; community care providers, including paediatricians, nursing teams and therapy services; and specialist care providers, such as neonatologists and hospital teams. Three categories were generated from the data: parent support needs (related to early identification and intervention), disintegrated care and healthcare professional experience and behaviours.

### Parent support needs

Parents reported that the unexpected preterm birth, associated psychological distress caused by risks to their child’s life and health, and separation from their baby created a unique context for becoming parents. This disrupted the anticipated natural start to parenthood and introduced the possibility of long-term medical and caregiving demands:

You just do not think it’s going to happen, especially when you have a pregnancy with no problems, you think you’re going to get to the full term. (23)

The development of an identity as parents of children with complex medical needs introduced unforeseen responsibilities and heightened vigilance regarding their child’s health and developmental progress. The awareness that their child had distinct needs compared with full-term infants served as a constant reminder of the lasting impact of preterm birth:

It’s like another reminder that your child is not normal…. she’s not going to have the same kinds of [developmental] milestones as all these other babies. (24)

Parents identified uncertainty as a primary factor influencing their support needs, emphasising the need for psychological support to manage this. Initially, the uncertainty was existential and concerned the child’s survival; however, during the child’s first year, diagnostic and prognostic uncertainties became challenging. Monitoring developmental milestones and undertaking practical interventions to address deviations from what they or medical professionals expected became an integral part of their responsibilities:

I always say, the first year of his life, I was more just his carer than his mum in terms of in and out of hospital, doing the exercises, just getting them to do stuff for him. There was no joy. (15)

Parents of children with known increased risk of neurodisability (such as brain injury seen on a scan) reported an early sense that something was different about their child. They preferred openness and early diagnosis, which validated their observations and legitimised accessing support. They also acknowledged that some HCPs appeared conflicted about providing such diagnoses early due to the evolving nature of child development:

There have been hints in the past that that [cerebral palsy] is the diagnosis, and we knew that would be the diagnosis, which is why we pushed for it. I felt whenever we had appointments, there was an elephant in the room. (08)

Parents emphasised that receiving a diagnosis did not eliminate uncertainty; instead, it introduced a new form of prognostic uncertainty, including how to manage the care of a child with complex medical needs while maintaining hope for the future.

So, we’ve just decided whatever the boys’ situation is, we’ll just cross that bridge when we come to it…what needs to be done here today and try not to worry too much about what might or might not be”. (05)

Parents also highlighted that the priorities of developmental follow-up services focused exclusively on their child, lacking an opportunity to address their own psychological needs.

At these clinics, I felt like going, going, “Actually, this is not about her, this is about me. And I’m not okay. I don't know how long I can do this. (01)

Where psychological support was available, parents valued it highly, although they reported that third-sector organisations predominantly provided these services. When continuity of care existed between inpatient and community settings, it became particularly beneficial, eliminating parents’ need to repeatedly explain their experiences. Importantly, this removed the need for parents to seek support: regular check-ins enabled help when needed and provided an individualised approach that considered caregiving and family commitments.

[Named charity] offered a lot of support. I would speak to her [psychologist] every week, and she was brilliant. At first, I was like, “No, I don’t need this. I’m fine.” Then, I thought, “Hang on a second, it’s probably better to process it properly.” Some weeks, when I was fine, I wouldn’t see her, but she would ring me every three weeks. (21)

Parents placed importance on the ability to connect with others who faced similar experiences. This helped validate their feelings, offered informational support and provided a perspective on the future. This community building also reduced isolation and helped parents develop long-term support systems beyond what healthcare services could offer.

There’s people [other parents] there that are ahead of you with things and there’s people that have just started the journey, so you all help each other out. (06)

### Disintegrated care

Parents described how the responsibility for ongoing care after discharge home moved from one to multiple service providers, each operating within different layers of healthcare organisations and systems. This complexity made it challenging for them to identify the right contact for support:

When we left [the hospital], it was really difficult. We didn’t know who dealt with what. (03)

This was especially evident for families who experienced additional structural vulnerabilities. Parents who lived geographically far from specialist services and had no access to private transport or low income had to navigate additional and unequal challenges to attend appointments:

It’s literally just a case of in, weigh him, the doctor just looks at him and he’s like, “Yeah, everything’s fine. See you in six months.” Literally 10 minutes. We have to get a taxi. It’s about £15 there and £15 back. So that’s a lot of money. (13)

Parents were often unsure which HCP or service had primary responsibility for their child’s care, requiring them to navigate multiple services and systems. This coordination role was burdensome, requiring them to understand complex systems, relationships and rules to determine their child’s follow-up care entitlement and how to access it.

You're passing the hot potato. How can my little girl have such a big intervention team, she’s surrounded by everybody, but yet seen by nobody. I’m trying to plug all these gaps. (15)

[Hospital 1] were like, it’s local [hospital 2] follow-up. So, I felt like we didn’t fit in either. It was a very stressful time. (02)

When accessing community therapy services, they expressed frustration and felt compelled to be assertive in efforts to secure adequate care. Barriers such as the need for specific referrals or diagnoses and lengthy waiting times obstructed access to care.

It’s taken three referrals for occupational therapy to get him put on the list. I’ve had to go to PALS^1^ twice for that, to get him on the list. The only reason they’ve accepted him now is because he’s been given a diagnosis. I don’t feel like I should’ve had to fight so much. (01)

Parents also reported that they believed their child may have ‘caught up’ on developmental milestones by the time they reached 2 years of age. This narrative, along with the reported expectation that their child would be discharged from neonatal services at 2 years of age, was interpreted by some as a sign that their child would have moved beyond the risks associated with prematurity.

So, they say at two years, they should be at their full….they should be kind of caught up is what they say. (19)

### Healthcare professionals' experience and behaviours

Parents described an evolving need for information during their child’s first 2 years, particularly around breathing, feeding and developmental progress. They wanted practical guidance on supporting their child’s changing needs at each developmental stage.

If there’s something, like a guide, to say, 'This is what you might- prepare yourself for’ or ‘This is what a baby looks like or does at that stage.’ I think that would have gone a long way. (11)

While these needs also resemble those of parents of term-born children, the circumstances for parents of preterm-born children involve increased complexity and uncertainty, intensifying the need for support.

Parents reported receiving contradictory information from HCPs across multiple services, causing increased anxiety and a lack of trust. They found it difficult to remember when to use their child’s chronological or corrected age for developmental milestones, readiness to start solid foods and vaccination timing. They emphasised that health visitors were not equipped to advise them on the specific needs of premature babies.

It was the one-year check. I just said, “They won’t even be eight months corrected and you’re going to come out and do a twelve-month check?”. She was like, “I’ll just go through it anyway.” “Can they crawl?” “No.” “Can they do this?” “No.” “Oh, there’s loads of red. (12)

When parents' information needs were unmet, they were likely to seek information from alternative sources, including peers, online and third-sector resources. They emphasised that being directed to trusted and reliable information sources was beneficial.

Some kind of guidance would be very helpful. I got told to do things on the ward, but then nothing. I found it myself; I’ve gone and looked stuff up. (02)

They also reported difficulty interpreting written developmental clinic documentation, highlighting that the composite scores generated from detailed developmental assessments were difficult to understand and did not meaningfully capture their child’s lived experience.

You could look at all the diagnoses and all the ASQ and Bayley [Ages and Stages Questionnaires and Bayleys’s Scales of Infant aand Toddler Development] levels they’ve got, but when you see them, they’re just happy. That’s what we felt was lacking from the paediatrician appointment, what they can do and the way they are, there’s so much more to them. (05)

**Figure 2:**
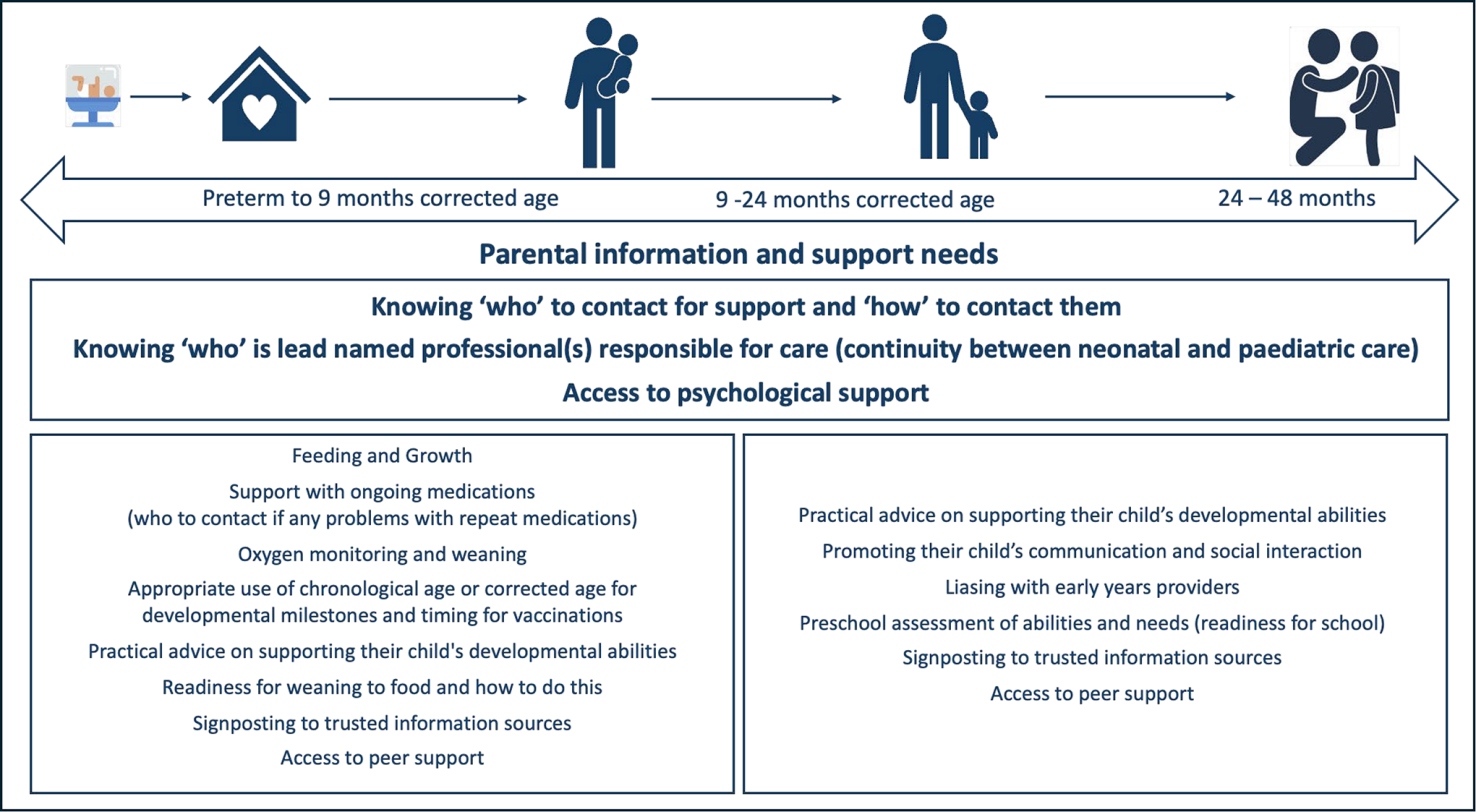
Parent information needs for preterm developmental follow-up.

Figure 2 summarises the information and support needs identified by parents at different timepoints.

Developing trusted relationships with specific clinicians was highly important. The healthcare worker’s profession and the organisation or system in which they worked were inconsequential; rather, what mattered was the individual’s availability and accessibility, as well as their capacity to understand the family’s journey and offer support. This involved opportunities to reflect, allowing parents to evaluate appointments and to gain further information. Parents also recognised the need for interactions with HCPs with expertise in prematurity to provide targeted, evidence-informed interventions and support should further difficulties emerge:

After the appointment, I’d put my concerns over to her, and she’d then go and maybe speak to dieticians or the neonatal consultants and then get back to me. (09)

If I hadn’t had that 12-week review with them [neonatal MDT clinic] to do those videos, I probably wouldn’t have realised there was anything wrong. (20)

## Discussion

This study aimed to understand the experiences of parents of extremely preterm infants with neonatal developmental follow-up care. Parents understood developmental follow-up as a process focused on their child’s health, growth and development, involving interactions with a diverse range of HCPs across different service providers. The findings highlight the challenges they face in managing post-discharge care for their child’s health and developmental needs, alongside their own emotional needs. They highlight the need for changes in care priorities, including a family-systems approach that incorporates parental mental well-being, training for universal healthcare providers and the development of integrated systems across service providers and geographic areas.

Parents expressed that insufficient psychological support was available following discharge home. Among parents of infants requiring neonatal care, there is a high prevalence of mental health challenges, such as anxiety and post-traumatic stress disorder (PTSD), within the first year of the infant’s life.^18 19^ These rates are significantly higher than the prevalence of anxiety observed among women during the perinatal period^20 21^ and among women in the general population.^22 23^ Most study participants were mothers, likely due to their greater availability during maternity leave, a pattern that aligns with existing gender norms.^24^ Theoretical sampling contributed to the inclusion of fathers, ensuring that the findings also reflected broader family dynamics. A family systems approach views the family as an interdependent system and reframes the idea that the infant is the sole focus of follow-up care. Instead, it emphasises the system within which the infant develops, an evolving relational system, is influenced by the social and cultural contexts in which the family is situated.^25^

Previous research supports the need for clear, consistent information from HCPs regarding the child’s current developmental abilities and prognosis: conflicting reports from different professionals can add to parental anxiety.^26–28^ Parents also need to understand the methods and timing of tools used to assess growth, health and developmental progress. Confusion about what is evaluated at different stages and about whether chronological or corrected age should be used to assess developmental abilities significantly increases parental burden. Specialist developmental follow-up services reportedly operate in parallel with, or independently of, universal child health programmes, rather than functioning together as integral components of a cohesive system.

Experiences described by parents are similar to those of parents whose children have complex medical needs.^29–31^ Complex care is a specialised field that coordinates care for children with complex medical conditions involving multiple providers.^32^ Although being born extremely preterm is not a chronic condition itself, it is a risk factor for lifelong health conditions and developmental challenges.^33–35^ Children born preterm are more likely to have ongoing medically complex care needs.^36^ The ‘liminal space’ between being told their child is ‘high risk’ of lifelong medically complex needs and receiving a diagnosis for specific conditions can contribute to uncertainty about the future and practically make it more difficult to access health services.^37^ There is considerable evidence that parents value early identification and early comprehensive diagnostic assessment.^38^

The NICE clinical guideline positions enhanced developmental follow-up as an integral part of neonatal services and recognises that neonatal services should work collaboratively with universal child health services to deliver this. It also advises that parents of preterm infants who have concerns about their child’s development should be directed to their GP or health visitor.^5^ In recommending this, the guideline assumes that universal and community healthcare providers have the knowledge and expertise to fulfil this role, without explicitly addressing whether their training prepares them to do so. Our findings indicate that this level of expertise does not reliably exist, creating a gap between what the guideline recommends and what is being delivered in practice.

Given advancements in neonatal care and growing evidence highlighting the need for extended care,^33 35^ historically established and arbitrary time points and metrics, initially designed to encompass the scope of neonatal care and outcomes, may no longer be fit for purpose. Some parents are unaware that their child continues to face elevated risks of health and developmental difficulties associated with preterm birth after 2 years of age, and evidence indicates that follow-up past preschool age rarely occurs, with only 6% of children and children born extremely preterm having a 4-year review.^16^ Without a clear integration pathway to paediatric care or continued follow-up beyond 2 years, discharge from neonatal services may serve as implicit reassurance that everything is fine. It may be better to acknowledge that the absence of difficulties for a child at 2 years of age is a positive indication of progress, but that health and developmental challenges related to prematurity may still emerge over time. Care that extends beyond 2 years of age is needed, and responsibility for this should be maintained across service boundaries so the receiving service is prepared for the child before the originating service ceases.

What parents consistently describe needing regarding their child’s health, growth, and developmental follow-up is, in principle, relatively straightforward: they want to be supported by HCPs who understand their child’s journey and have the experience and expertise to advise them appropriately. This may be a thread of continuity, a named person, or an integrated pathway to community and paediatric services. Families facing structural vulnerabilities^39^ related to health literacy, income inequality, or geographic isolation encounter additional cumulative barriers to care. These may include additional demands, such as managing administrative tasks to access services, arranging transportation to reach services, and relying on interpreters for communication, which can further diminish the consistency of care. The impact of these challenges will be most severe for families lacking sufficient structural support to manage them.

### Strengths and limitations

A strength of this study was that data were collected from three diverse neonatal services, using purposive sampling to select sites varying in size, organisational structure and level of neonatal inpatient care. Potential participants were also chosen to represent a broad range of experiences. Potential interviewees were offered a variety of interview approaches to minimise the burden of participation. Data collection and analysis were undertaken iteratively and reflexively, with the primary researcher (CM) supported by a study supervisory team and parent (lived experience) advisory group, recognising that the findings represent a construction between the researcher and participants.

Limitations include the exclusion of non-English-speaking parents, which may mean the findings do not represent those most affected by health inequities such as those experiencing structural barriers related to health literacy. Additionally, only parents of infants born less than 30 weeks were included, as this group is specifically highlighted for enhanced developmental surveillance and support in the NICE clinical guideline. We recognise that children born moderately preterm also face a higher risk of adverse neurodevelopmental outcomes. Future research should continue to explore fathers’ and broader family experiences.

## Conclusions

The challenges to children’s health and development caused by premature birth persist through infancy, childhood, adolescence, and into adulthood. The impact of these profoundly affects parental well-being, as well as individuals born preterm. Neonatal follow-up should be an integral part of care, considering the child’s health, growth and development, as well as parental well-being. A shift from reactive, episodic neonatal follow-up to a family-focused, proactive, population healthcare model across healthcare systems is needed. This could include acknowledging the work parents do in caring for their child; routinely supporting parental mental health; evaluating parental capacity and social determinants; and refocusing policy from clinical data collection toward a model that emphasises family and child functioning and outcomes important to families.^40^ Universal child health providers need more training in caring for premature children and their families, and community therapy services need to adopt a proactive approach to accepting referrals, based on sound evidence that those at ‘high risk’ receive timely support and intervention, rather than requiring specific referrals or diagnoses to access services.

## Data Availability

Data are available upon reasonable request.

## Footnotes

PALS, the NHS Patient Advice and Liaison Service is a free confidential service in hospitals that offer support and information to families and career. They help to resolve concerns and can advise on making formal complaints.
