# Supplementary material for: ‘She’s surrounded by everybody, but yet seen by nobody.’ Parents’ experiences and priorities for neonatal developmental follow-up care
Source: BMJ Paediatr Open. 2026 Aug 7;10(1):e004772. doi: 10.1136/bmjpo-2026-004772 (PMC13475087; doi:10.1136/bmjpo-2026-004772)
Supplement: Supplementary file 1 [file bmjpo-2026-004772.reviewer_comments.pdf]

PEER REVIEW HISTORY

BMJ Paediatrics Open publishes all reviews undertaken for accepted manuscripts. Reviewers are asked to complete a checklist review form and are provided with free text boxes to elaborate on their assessment. These free text comments are reproduced below.

ARTICLE DETAILS

**TITLE (PROVISIONAL)**	"She's surrounded by everybody, but yet seen by nobody". Parents' experiences and priorities for neonatal developmental follow-up care.
**AUTHORS**	Marcroft, Claire<br>Basu, Anna Purna<br>Kolehmainen, Niina<br>Thomson, Richard<br>Exley, Catherine

VERSION 1 – REVIEW

**REVIEWER NAME**	Pinzón-Segura, María
**REVIEWER AFFILIATION**	Universidad Nacional de Colombia
**REVIEWER CONFLICT OF INTEREST**	No Conflict of Interest
**AI DISCLOSURE**	This review was developed with the assistance of ChatGPT (OpenAI) as a language support and drafting tool. The final analysis, critical appraisal, interpretations, and recommendations were independently reviewed, edited, and validated by the reviewer.
**DATE REVIEW RETURNED**	26-May-2026

**GENERAL COMMENTS**	This manuscript addresses a highly relevant and underexplored area in neonatal developmental follow-up care, with important implications for pediatrics, public health, and health systems organisation. The study provides a valuable qualitative contribution by shifting attention beyond survival and neurodevelopmental outcomes toward parental experiences, care coordination, psychosocial burden, and continuity of care after discharge.<br><br>The introduction is well developed and clearly contextualises current NICE recommendations, neonatal operational delivery networks, and existing gaps in post-discharge follow-up pathways. The use of Constructivist Grounded Theory is methodologically appropriate and coherent with the study aims. The qualitative rigor is strengthened by concurrent analysis, memoing, reflexivity, purposive/theoretical sampling, peer debriefing, and meaningful parent advisory group involvement.<br><br>The three major categories generated — parental support needs, disintegrated care, and healthcare professional experience and behaviours — are conceptually strong and supported by rich participant quotations. One of the manuscript's greatest strengths is its systems-level interpretation of the problem. Rather than individualising responsibility, the findings identify fragmentation across services, unclear accountability, and lack of integration between neonatal, community, and universal child health systems.<br><br>The manuscript also makes an important contribution by highlighting

	unmet parental psychological needs following discharge. The findings strongly support the need for a family-focused and proactive model of neonatal developmental follow-up care. Particularly important is the observation that discharge from neonatal services at two years may unintentionally reassure families despite the persistent risk of later-emerging developmental difficulties.<br><br>From an epidemiological and health services perspective, the study effectively reframes developmental follow-up as a longitudinal systems issue rather than solely a child-level clinical outcome. The findings also have strong potential to inform future mixed-methods and implementation research, as several identified dimensions — including parental care coordination burden, uncertainty, continuity of care, and trust in providers — could later be operationalised quantitatively.<br><br>Several areas could be strengthened. First, although the exclusion of non-English-speaking parents is acknowledged, this limitation deserves deeper discussion. Families experiencing migration, racialisation, lower health literacy, poverty, or other forms of structural vulnerability may experience even greater fragmentation in care. Given the systems-oriented framing of the manuscript, incorporating perspectives related to health inequities, structural vulnerability, social determinants of health, and differential access to services would substantially strengthen the discussion.<br><br>Second, the predominance of maternal participants limits exploration of paternal experiences, coparenting dynamics, and broader family perspectives. While common in neonatal research, the implications of this gender imbalance for category construction and interpretation should be discussed more explicitly.<br><br>Third, the manuscript appropriately adopts the concept of “theoretical sufficiency” within contemporary Constructivist Grounded Theory; however, additional clarification regarding how sufficiency was determined would improve methodological transparency. In particular, it would be useful to describe when new conceptual properties ceased to emerge, how categories stabilised, and which analytic decisions supported sample closure.<br><br>Fourth, the study includes children across a relatively broad developmental range (0–32 months corrected age), yet parental experiences and support needs likely evolve substantially over time. Greater temporal analysis comparing earlier versus later experiences, or exploring how uncertainty changes across developmental stages, could further enrich the conceptual model.<br><br>Finally, although the discussion refers to a “family systems approach,” this concept could be developed more theoretically through stronger linkage with family-centred care, trauma-informed care, family systems theory, or ecological developmental frameworks.<br><br>Overall, this is a strong, clinically meaningful, and methodologically rigorous manuscript with important implications for neonatal follow-up policy and service design. I believe the manuscript is suitable for publication following minor to moderate revisions focused primarily on expanding the discussion around health inequities, conceptual framing, and methodological clarification.

REVIEWER NAME	**Raman, Shanti**
**REVIEWER AFFILIATION**	South Western Sydney Local Health District, Department of Community Paediatrics
**REVIEWER CONFLICT OF INTEREST**	No Conflict of Interest
**AI DISCLOSURE**	No, I have not used AI
**DATE REVIEW RETURNED**	10-Jun-2026

GENERAL COMMENTS	General<br>This is a really important topic and I commend the authors for tackling it. It is a well conceptualised and executed qualitative research study, with the main author/researcher demonstrating excellent reflexivity. Beautiful study, very little to critique.<br>In the Key Messages- "What this study adds: please say:<br>Parents understand developmental follow-up as a process that focuses on their child's health, growth, and development, involving interactions with diverse health professionals, but did prefer to have early diagnostic confirmation of neurodevelopmental conditions associated with prematurity.<br>Methods<br>Authors highlight that these were diverse NHS service providers/settings- but did they look for diversity in the participants? Were any of the families from non-English speaking backgrounds? This might need to be explained. Whilst this is mentioned in the Limitations section in the Discussion, I feel it needs to be stated in the Methods right up front.<br>Results<br>I think that Theme 1 should be more tailored to the specific research questions- which is about neurodevelopmental follow up. So perhaps rather than "Parent support needs"- which is fairly generic and yes all parents need support following the birth of a premature infant- I would title it: "Parental support needs related to early diagnosis and intervention". I think this is an important finding and needs to be elevated in your reporting and summary: Parents expressed a preference for the early diagnosis of specific conditions associated with preterm birth.<br><br>Discussion<br>Generally well done.<br>Paragraph 4- when authors state this: "The 'liminal space' between being told their child is 'high risk' of lifelong medically complex needs and receiving a diagnosis for specific conditions, can contribute to uncertainty about the future, and practically make it more difficult to access health services." Please also add "There is considerable evidence from the literature that parents value early identification and early comprehensive diagnostic assessment."

VERSION 1 – AUTHOR RESPONSE

Reviewer: 1
Dr. María Pinzón-Segura, Universidad Nacional de Colombia

Comments to the Author:
This manuscript addresses a highly relevant and underexplored area in neonatal developmental follow-up care, with important implications for pediatrics, public health, and health systems organisation. The study provides a valuable qualitative contribution by shifting attention beyond survival and neurodevelopmental outcomes toward parental experiences, care coordination,

psychosocial burden, and continuity of care after discharge.

The introduction is well developed and clearly contextualises current NICE recommendations, neonatal operational delivery networks, and existing gaps in post-discharge follow-up pathways. The use of Constructivist Grounded Theory is methodologically appropriate and coherent with the study aims. The qualitative rigor is strengthened by concurrent analysis, memoing, reflexivity, purposive/theoretical sampling, peer debriefing, and meaningful parent advisory group involvement.

The three major categories generated — parental support needs, disintegrated care, and healthcare professional experience and behaviours — are conceptually strong and supported by rich participant quotations. One of the manuscript's greatest strengths is its systems-level interpretation of the problem. Rather than individualising responsibility, the findings identify fragmentation across services, unclear accountability, and lack of integration between neonatal, community, and universal child health systems.

Corresponding author response: Thank you for your thoughtful feedback and for confirming the important implications for this work. In addition, we thank you for sharing your methodological expertise and the positive feedback about the appropriateness of the methodological approach and qualitative rigour. We are pleased that our reporting of these data highlights the systemic challenges to neonatal developmental follow-up, as this concept is grounded in the data.

The manuscript also makes an important contribution by highlighting unmet parental psychological needs following discharge. The findings strongly support the need for a family-focused and proactive model of neonatal developmental follow-up care. Particularly important is the observation that discharge from neonatal services at two years may unintentionally reassure families despite the persistent risk of later-emerging developmental difficulties.

From an epidemiological and health services perspective, the study effectively reframes developmental follow-up as a longitudinal systems issue rather than solely a child-level clinical outcome. The findings also have strong potential to inform future mixed-methods and implementation research, as several identified dimensions — including parental care coordination burden, uncertainty, continuity of care, and trust in providers — could later be operationalised quantitatively.

Several areas could be strengthened. First, although the exclusion of non-English-speaking parents is acknowledged, this limitation deserves deeper discussion. Families experiencing migration, racialisation, lower health literacy, poverty, or other forms of structural vulnerability may experience even greater fragmentation in care. Given the systems-oriented framing of the manuscript, incorporating perspectives related to health inequities, structural vulnerability, social determinants of health, and differential access to services would substantially strengthen the discussion.

Corresponding author response: Thank you for this constructive observation. We have added data in the finding's sections (lines 436-442) and expanded the discussion (lines 658-664) to address this

Second, the predominance of maternal participants limits exploration of paternal experiences, coparenting dynamics, and broader family perspectives. While common in neonatal research, the implications of this gender imbalance for category construction and interpretation should be discussed more explicitly.

Corresponding author response: Thank you for highlighting this. We have added text in the discussion to recognise that most participants were mothers, but that theoretical sampling contributed to the inclusion of fathers to ensure findings reflected broader family dynamics (lines 574-577)

Third, the manuscript appropriately adopts the concept of “theoretical sufficiency” within contemporary Constructivist Grounded Theory; however, additional clarification regarding how sufficiency was determined would improve methodological transparency. In particular, it would be useful to describe when new conceptual properties ceased to emerge, how categories stabilised, and which analytic decisions supported sample closure.

Corresponding author response: Thank you. The analytic decisions supporting sample closure (sufficiency) were made when the defining characteristics of the categories were clearly expressed (had stabilised) and consistently appeared in parents’ narratives with confirmation by the parent advisory group supporting sample closure. Clarity about this has been included in the methods (lines 182-192)

Fourth, the study includes children across a relatively broad developmental range (0–32 months corrected age), yet parental experiences and support needs likely evolve substantially over time. Greater temporal analysis comparing earlier versus later experiences, or exploring how uncertainty changes across developmental stages, could further enrich the conceptual model.

Corresponding author response: Thank you for highlighting this. We have added context on how parents identified uncertainty as a primary factor influencing their support needs, emphasising the need for psychological support to manage this, and how this uncertainty changes over time (but does not entirely resolve). Lines 337-350.

Finally, although the discussion refers to a “family systems approach,” this concept could be developed more theoretically through stronger linkage with family-centred care, trauma-informed care, family systems theory, or ecological developmental frameworks.

Corresponding author response: Thank you for this suggestion. We have developed this further in lines 399-402

Overall, this is a strong, clinically meaningful, and methodologically rigorous manuscript with important implications for neonatal follow-up policy and service design. I believe the manuscript is suitable for publication following minor to moderate revisions focused primarily focused on expanding the discussion of health inequities, conceptual framing, and methodological clarification.

Corresponding author response: Thank you, we hope our response and amendments meet your requirements and address the important points you have identified.

<u>Reviewer: 2</u>

Dr. Shanti Raman, South Western Sydney Local Health District, University of New South Wales

General
This is a really important topic and I commend the authors for tackling it. It is a well conceptualised and executed qualitative research study, with the main author/researcher demonstrating excellent reflexivity. Beautiful study, very little to critique.

Corresponding author response: Thank you for your positive comments.

In the Key Messages- “What this study adds: please say:
Parents understand developmental follow-up as a process that focuses on their child’s health, growth, and development, involving interactions with diverse health professionals, but did prefer to have early diagnostic confirmation of neurodevelopmental conditions associated with prematurity.

Corresponding author response: This has been added on lines 53-55

Methods

Authors highlight that these were diverse NHS service providers/settings- but did they look for diversity in the participants? Were any of the families from non-English speaking backgrounds? This might need to be explained. Whilst this is mentioned in the Limitations section in the Discussion, I feel it needs to be stated in the Methods right up front.

Corresponding author response: Thank you. This has been included more explicitly in the methods section (sampling and recruitment). Specifically, lines 139-140, 161 and the participant characteristics section 229-235.

Results

I think that Theme 1 should be more tailored to the specific research questions- which is about neurodevelopmental follow up. So perhaps rather than "Parent support needs"- which is fairly generic and yes all parents need support following the birth of a premature infant- I would title it: "Parental support needs related to early diagnosis and intervention". I think this is an important finding and needs to be elevated in your reporting and summary: Parents expressed a preference for the early diagnosis of specific conditions associated with preterm birth.

Corresponding author response: Thank you. We have amended this to highlight the focus on needs related to early identification and intervention. We discussed this with our parent advisory group, and they felt that it is true that parents should have support following the birth of a premature infant, but most don't receive this. We have added your suggestion to the summary of categories, but kept the category more broadly 'parent support needs'.

Discussion

Generally well done.

Paragraph 4- when authors state this: "The 'liminal space' between being told their child is 'high risk' of lifelong medically complex needs and receiving a diagnosis for specific conditions, can contribute to uncertainty about the future, and practically make it more difficult to access health services." Please also add "There is considerable evidence from the literature that parents value early identification and early comprehensive diagnostic assessment."

Corresponding author response: This has been added in lines 614-616

<u>Associate Editor:</u>

Thank you for the opportunity to review this manuscript. This is a timely and important qualitative study addressing parental experiences of neonatal developmental follow-up after very preterm birth. The manuscript focuses on a clinically and policy-relevant gap: while survival among infants born at the earliest gestational ages has improved, corresponding improvements in neurodevelopmental outcomes have not been clearly demonstrated, and post-discharge care pathways remain less developed than antenatal and acute neonatal pathways.

Overall, the manuscript has several strengths. The research question is relevant, the qualitative material is rich, and the findings provide a meaningful account of parents' unmet psychological needs, experiences of fragmented care, and concerns about inconsistent professional knowledge across services. The use of Constructivist Grounded Theory is appropriate, and the involvement of a parent advisory group strengthens the credibility and relevance of the analysis. However, I recommend minor revision because several aspects require further development before the manuscript can fully support its conclusions and meet expectations for transparent qualitative reporting.

Corresponding author response: Thank you for your positive comments about this important work. We hope you find our responses suitable for the minor revision.

The manuscript states that reporting follows the COREQ guidelines. However, several COREQ-relevant details remain insufficiently reported. The authors describe the qualitative design, the use of semi-structured interviews, concurrent data generation and analysis, memo writing, reflexivity, and parent advisory group involvement. These are important strengths. Nevertheless, the manuscript would benefit from a more explicit account of key reporting items.
In particular, the authors should clarify: How many parents were eligible, approached, declined participation, or did not respond. Whether any non-participants differed meaningfully from participants, if this information is available. Whether anyone else was present during interviews. Whether transcripts, preliminary findings, or themes were returned to participants for comment. How the movement from initial codes to focused codes and final categories was documented and reviewed.

Corresponding author response: Thank you for this attention to detail. The transcripts, preliminary findings, or themes were not returned to participants for comment; however, the parent advisory group, comprising 4 individuals with lived experience, met bimonthly throughout the study and reviewed manuscripts and preliminary findings, and contributed to the development of codes and final categories. This is documented in lines 181-192.

The manuscript currently states that recruitment continued until theoretical sufficiency was reached, but the analytic basis for this decision is not sufficiently transparent. The authors should explain which categories had become conceptually coherent, what variation was still being explored, and how later interviews confirmed, extended, or challenged emerging categories.

Corresponding author response: The analytic decisions supporting sample closure (sufficiency) were made when the defining characteristics of the categories were clearly expressed (had stabilised) and consistently appeared in parents' narratives with confirmation by the parent advisory group supporting sample closure. Clarity about this has been included in the methods (lines 181-192).

The inclusion and exclusion criteria are clearly listed. However, the rationale for some exclusions requires further explanation. The authors excluded parents unable to consent, parents of infants diagnosed with or suspected to have genetic disorders, and parents who could not speak English. These decisions may be methodologically defensible, but the manuscript does not yet explain them adequately. The exclusion of non-English-speaking parents is especially important, given that the study examines fragmented care, access to support, health literacy, and inequities in post-discharge services. The limitation section acknowledges that excluding non-English-speaking parents may omit those most affected by health inequities, structural barriers, or different cultural models of illness. This is useful, but the decision also needs to be justified in the methods section. The authors should clarify whether this exclusion was due to funding, translation capacity, ethical constraints, or analytic scope.

Corresponding author response: Exclusion was due to translational capacity, but we did include families whose first language was not English. We have added these data in lines 229-230 of the findings section and have expanded the discussion (lines 658-664) to address these important points.

The exclusion of genetic disorders also needs a clearer rationale. If the purpose was to focus specifically on prematurity-related developmental follow-up and avoid additional etiological complexity, this should be stated. However, the authors should also acknowledge that this exclusion may remove families with particularly complex follow-up trajectories, whose experiences could be highly relevant to the study's concern with service fragmentation.

Corresponding author response: The aim of the study was to focus on parents' experience of neonatal follow-up care following preterm birth less than 30 weeks gestation (as advised by UK clinical guidelines). The group of patients with genetic disorders would receive a different pathway of care than those born preterm, and there is parallel research in optimal pathways for children with medical complexity and their families, which would cover this group of families with children with genetic disorders.

The phrase "parents unable to consent" also requires clarification. The manuscript reports that two parents had physical or learning disabilities affecting their ability to care for their baby. This makes it important to specify how capacity to consent was assessed and whether reasonable adjustments were offered to support participation where appropriate.

Corresponding author response: Thank you. One parent was supported by his partner. One parent had a congenital hand difference and made adaptations to independently care for their baby. Both parents clearly met the criteria for capacity to consent, and reasonable adjustments were offered to support participation where appropriate. This has been updated in lines 229-233.

The manuscript uses Constructivist Grounded Theory as a methodological approach, but the theoretical framing of the substantive problem remains underdeveloped. The introduction is currently grounded mainly in clinical guidelines, neonatal service organisation, and evidence on developmental risk. This is necessary, but not sufficient for a qualitative study focused on parental experience, care fragmentation, and family needs.
This theoretical strengthening would also help connect the findings to the manuscript's policy implications. For example, the concept of care navigation burden would directly support the finding that parents struggled to identify who was responsible for their child's care and often had to "fight" for access to services.

Corresponding author response: Thank you for highlighting this. We have added this in the introduction (lines 92-95 and 106-110) with appropriate references

The conclusion argues for a shift from reactive, episodic neonatal follow-up to a family-focused, proactive, preventative population health model. This is an important claim, but it requires more operational detail. The manuscript should specify what such a model would involve in practice.

Corresponding author response: We have added more specific details and citations in the discussion (lines 577-581) and conclusion (690-694)

This manuscript addresses an important gap in neonatal follow-up care and presents valuable qualitative findings with clear implications for clinical practice, service organisation, and policy. However, before publication, the authors should strengthen the methodological reporting, justify the exclusion criteria more explicitly, and develop a stronger theoretical and conceptual integration of the findings. These revisions would allow the manuscript to move beyond a descriptive account of parental experience and make a clearer contribution to understanding neonatal developmental follow-up as a family-centred, integrated, longitudinal system of care.

Corresponding author response: Thank you. We have addressed these.

VERSION 2 – REVIEW

**REVIEWER NAME**	Pinzón-Segura, María
**REVIEWER AFFILIATION**	Universidad Nacional de Colombia

**REVIEWER CONFLICT OF INTEREST**	No Conflict of Interest
**AI DISCLOSURE**	I used OpenAI's ChatGPT (based on the GPT-4 architecture) as a language support tool during the preparation of my review. Its role was limited to assisting with tasks such as refining the language, clarifying and organizing my thoughts, and ensuring the text adhered to a clear and structured format. All substantive evaluations and the ultimate conclusions expressed in the review reflect my own critical assessment, and the AI served solely as a writing aid.
**DATE REVIEW RETURNED**	06-Jul-2026

**GENERAL COMMENTS**	I appreciate the authors' careful and thoughtful revision of the manuscript. They have addressed all of my previous comments as well as those raised by the second reviewer and the Associate Editor. The revised manuscript is clearer, methodologically stronger, and provides a more balanced discussion of the implications of the findings.<br><br>The authors have substantially improved several aspects of the manuscript, including:<br>* greater methodological transparency regarding theoretical sufficiency and sample closure;<br>* improved reporting consistent with COREQ recommendations;<br>* clearer description of participant diversity;<br>* expanded discussion of health inequities and the exclusion of non-English-speaking families;<br>* stronger conceptual framing around family-centred care and parental psychological needs;<br>* clarification regarding the evolving nature of uncertainty throughout developmental follow-up;<br>* strengthening of the "What this study adds" section and overall presentation.<br><br>These revisions considerably strengthen the manuscript without altering its original contribution.<br><br>Remaining minor observations, that does not justify a further round of revisions (do not affect the validity or overall quality of the work), but<br>should be included:<br>1. Although the rationale for excluding non-English-speaking participants has been expanded, the justification in the Methods could still be stated more explicitly (e.g., lack of translation resources). At present, the explanation appears mainly in the response letter rather than being fully incorporated into the manuscript itself.<br>2. The manuscript continues to include predominantly maternal perspectives (18 mothers versus 2 fathers interviewed individually), and although this limitation is now acknowledged, future research should continue to explore fathers' and broader family experiences. The authors appropriately recognise this limitation.

**REVIEWER NAME**	Raman, Shanti
**REVIEWER AFFILIATION**	South Western Sydney Local Health District, Department of Community Paediatrics
**REVIEWER CONFLICT OF INTEREST**	No Conflict of Interest
**AI DISCLOSURE**	No, I have not used AI

DATE REVIEW RETURNED	07-Jul-2026

GENERAL COMMENTS	I am satisfied that the authors have thoughtfully responded to reviewers' comments and revised their submission accordingly.

VERSION 2 – AUTHOR RESPONSE

Reviewer: 1
Dr. María Pinzón-Segura, Universidad Nacional de Colombia
Comments to the Author
I appreciate the authors' careful and thoughtful revision of the manuscript. They have addressed all of my previous comments as well as those raised by the second reviewer and the Associate Editor. The revised manuscript is clearer, methodologically stronger, and provides a more balanced discussion of the implications of the findings.

The authors have substantially improved several aspects of the manuscript, including:
* greater methodological transparency regarding theoretical sufficiency and sample closure;
* improved reporting consistent with COREQ recommendations;
* clearer description of participant diversity;
* expanded discussion of health inequities and the exclusion of non-English-speaking families;
* stronger conceptual framing around family-centred care and parental psychological needs;
* clarification regarding the evolving nature of uncertainty throughout developmental follow-up;
* strengthening of the "What this study adds" section and overall presentation.

These revisions considerably strengthen the manuscript without altering its original contribution.

Remaining minor observations, that does not justify a further round of revisions (do not affect the validity or overall quality of the work), but
should be included:
1. Although the rationale for excluding non-English-speaking participants has been expanded, the justification in the Methods could still be stated more explicitly (e.g., lack of translation resources). At present, the explanation appears mainly in the response letter rather than being fully incorporated into the manuscript itself.

Authors Response: Thank you for your attention to detail. We have added this sentence in the exclusion criteria of the methods as recommended (lines 122-123)

2. The manuscript continues to include predominantly maternal perspectives (18 mothers versus 2 fathers interviewed individually), and although this limitation is now acknowledged, future research should continue to explore fathers' and broader family experiences. The authors appropriately recognise this limitation.

Authors Response: We have added this as an additional sentence in the strengths and limitations section to highlight this need (lines 446-447)

Reviewer: 2
Dr. Shanti Raman, South Western Sydney Local Health District, University of New South Wales
Comments to the Author

I am satisfied that the authors have thoughtfully responded to reviewers' comments and revised their submission accordingly.
